# Gut Microbiota and Dyslipidemia in Type 2 Diabetes: A Pilot Study of 16S rRNA Profiles and Predicted Functional Shifts

**DOI:** 10.1155/jdr/9317962

**Published:** 2026-04-02

**Authors:** Godfred Antony Menezes, Priyadharshini Sekar, Areebah Akhter, Ketaki Devendra Tayade, Sana Fathima, Zaina Falak Zahir Hussain, Abhay Nigam

**Affiliations:** ^1^ Department of Medical Microbiology and Immunology, Trinity Medical Sciences University School of Medicine, Kingstown, Saint Vincent and the Grenadines; ^2^ Department of Public Health and Epidemiology, College of Medicine and Health Sciences, Khalifa University, Abu Dhabi, UAE, kustar.ac.ae; ^3^ Microbiota Research Group, Research Institute of Medical and Health Sciences, University of Sharjah, Sharjah, UAE, sharjah.ac.ae; ^4^ Department of Medical Microbiology and Immunology, RAK College of Medical Sciences, RAK Medical and Health Sciences University, Ras Al Khaimah, UAE; ^5^ Specialist Physician, Al Zahrawi Hospital, Ras Al-Khaimah, UAE

**Keywords:** 16S rRNA sequencing, *Akkermansia muciniphila*, bile acids, *Collinsella*, dyslipidemia, Firmicutes/Bacteroidetes ratio, gut microbiota, KEGG pathways, PICRUSt2, short-chain fatty acids, type 2 diabetes mellitus

## Abstract

Hyperlipidemia is a major, modifiable driver of global cardiovascular risk. The intestinal microbiota, comprising bacteria, archaea, fungi, and viruses, modulates lipid metabolism through bile acid transformation, energy harvest, and inflammatory signaling. This study profiled the gut microbiota of 15 adults with type 2 diabetes mellitus (T2DM) and explored associations with fasting lipid measures using 16S rRNA gene sequencing (V3–V4 region) on the Illumina MiSeq platform and PICRUSt2 functional prediction. Overall α‐diversity was reduced, and community composition was dominated by Firmicutes and Actinobacteria with relative depletion of Bacteroidetes. At lower taxonomic ranks, enrichment of *Prevotella copri*, *Collinsella* spp., *Ruminococcus* spp., and selected *Bifidobacterium* spp. was observed, alongside depletion of short‐chain fatty acid (SCFA)‐linked taxa, including *Akkermansia muciniphila*, *Lactobacillus plantarum*, and members of the *Bacteroides* and *Parabacteroides* lineages. Exploratory within‐cohort trends indicated that higher triglycerides (TGs) and lower HDL‐C tended to co‐occur with increased *Collinsella* and clostridial signals and reduced SCFA‐associated taxa. Predicted Kyoto Encyclopedia of Genes and Genomes (KEGG) ortholog functions suggested shifts in lipid, carbohydrate, and secondary bile acid metabolism, consistent with a metabolically activated and proinflammatory intestinal milieu. In this single‐arm cohort of adults with T2DM, a low‐diversity, Firmicutes/Actinobacteria‐weighted microbiome with depletion of SCFA‐linked taxa paralleled an atherogenic lipid profile, supporting an association between gut microbial dysbiosis and lipid abnormalities in adults with T2DM. These findings suggest the potential of microbiota‐informed adjuncts, including dietary fermentable fiber, targeted probiotics and next‐generation biotherapeutics, and bile‐acid‐modulating strategies as supportive approaches to lipid management in T2DM. This was a pilot, single‐arm, exploratory study without a nondiabetic control group, and findings should be interpreted as hypothesis‐generating. Nevertheless, the cross‐sectional design, small sample size, and 16S‐based taxonomic resolution limit causal interpretation. Larger, longitudinal studies integrating shotgun metagenomics and metabolomics are needed to confirm these associations, validate biomarkers, and elucidate mechanistic pathways that could guide precision interventions for diabetic dyslipidemia.

## 1. Introduction

The human gastrointestinal tract harbors a dense and metabolically active microbial ecosystem exceeding 10^14^ microorganisms, encompassing bacteria, archaea, fungi, and viruses—collectively termed the gut microbiota. This community acts as a dynamic metabolic organ that regulates digestion, immune maturation, and host energy balance [1]. Beyond nutrient processing, the gut microbiome exerts endocrine‐like control over lipid absorption, bile acid transformation, and systemic lipid homeostasis [2].

Hyperlipidemia, defined by elevated plasma triglycerides (TGs), cholesterol, or low‐density lipoprotein (LDL), remains one of the leading modifiable risk factors for atherosclerotic cardiovascular disease worldwide [3]. While genetic predisposition, dietary habits, and physical inactivity contribute to its prevalence, increasing evidence implicates gut microbial dysbiosis as an important determinant of lipid metabolic disorders [4].

In a balanced or eubiotic state, commensal bacteria such as *Bacteroides* spp. and *Faecalibacterium prausnitzii* ferment dietary polysaccharides into short‐chain fatty acids (SCFAs), including acetate, propionate, and butyrate, that enhance epithelial barrier integrity, activate AMP‐activated protein kinase (AMPK) and peroxisome proliferator‐activated receptors (PPARs), and suppress hepatic lipogenesis [5, 6]. Through these mechanisms, SCFAs improve insulin sensitivity and maintain lipid homeostasis [2].

Conversely, dysbiosis characterized by the expansion of *Firmicutes*, *Ruminococcus*, and *Collinsella* species promotes intestinal permeability and lipopolysaccharide (LPS) translocation, triggering systemic inflammation and hepatic lipid accumulation [7]. Such microbial shifts enhance energy harvest and alter bile acid metabolism by modulating farnesoid X receptor (FXR) and Takeda G‐protein–coupled receptor 5 (TGR5) signaling pathways, thereby linking gut ecology to lipid dysregulation and insulin resistance [5, 6].

Multiple studies have reported that an increased Firmicutes/Bacteroidetes (F/B) ratio correlates positively with obesity, hypercholesterolemia, and hypertriglyceridemia [8]. Mechanistic investigations further demonstrate that microbial bile salt hydrolases (BSHs) mediate deconjugation of primary bile acids, influencing enterohepatic feedback on hepatic cholesterol synthesis and clearance [2]. However, most available data derive from Western and East Asian populations, and comparable datasets from other regions, where dietary lipid patterns and gut‐microbiota structures differ, remain limited [7].

Given this context, the present study characterizes gut microbial diversity, composition, and predicted metabolic functions in adults with type 2 diabetes mellitus (T2DM) exhibiting dyslipidemia. Using 16S rRNA gene sequencing on an Illumina MiSeq platform, Kyoto Encyclopedia of Genes and Genomes (KEGG) pathway inference, and correlation analyses with serum lipid profiles, this study aims to identify microbial taxa and functional signatures linked to lipid dysregulation. Understanding these host–microbe interactions may guide microbiota‐based dietary or probiotic interventions as adjunctive strategies for lipid management in T2DM [9, 10]. Accordingly, this study was designed as an exploratory pilot analysis to identify microbiota features that co‐occur with dyslipidemia in adults with T2DM, rather than to establish causality or disease‐specific signatures.

## 2. Materials and Methods

### 2.1. Study Design and Participants

This cross‐sectional study included 15 adults (11 males and four females) with clinically diagnosed T2DM. Participants were recruited from the Internal Medicine Department, Al Zahrawi Hospital, Ras Al Khaimah, United Arab Emirates, after providing written informed consent. All study procedures adhered to the ethical principles of the Declaration of Helsinki (2013) and were approved by the Institutional Ethics Committee of RAK Medical and Health Sciences University, Ras Al Khaimah, United Arab Emirates. Participant anonymity and data confidentiality were maintained throughout the study.

Each participant provided paired stool and fasting venous blood samples. Stool specimens were collected in sterile, DNA‐free containers for gut‐microbiome analysis, while venous blood was drawn by a qualified physician for biochemical and lipid profiling [11]. Lipid panels, including serum total cholesterol (TC), TGs, and high‐density lipoprotein cholesterol (HDL‐C), were quantified using standard enzymatic colorimetric assays (CHOD–POD and GPO–PAP methods) on an automated analyzer. Low‐density lipoprotein cholesterol (LDL‐C) was calculated using the Friedewald equation. None of the participants had used antibiotics, probiotics, prebiotics, or lipid‐modifying agents within the preceding 3 months.

Samples were transported under cold chain conditions (4°C) to preserve microbial viability. Stool aliquots were stored at −80°C until DNA extraction, and serum samples were stored at −20°C for biochemical assays.

Data on diabetes duration, glycated hemoglobin (HbA1c), detailed dietary intake, and long‐term medication exposure were unavailable and therefore could not be adjusted for in the analyses. These variables are known to influence both gut microbiota composition and lipid metabolism and represent potential sources of residual confounding.

### 2.2. DNA Extraction and Library Preparation

Fecal DNA was extracted using the QIAamp Fast DNA Stool Mini Kit (Qiagen, Germany) according to the manufacturer’s instructions. DNA concentration and purity were measured using a Qubit 4 Fluorometer (DNA HS Assay Kit, Thermo Fisher Scientific) and a NanoDrop Spectrophotometer (A260/280 ≈1.8). Extracts were stored in TE buffer at −80°C for long‐term stability.

Amplicon libraries targeting the V3–V4 hypervariable region of the 16S rRNA gene were amplified using primers 341F (5′‐CCTACGGGNGGCWGCAG‐3^′^) and 805R (5^′^‐GACTACHVGGGTATCTAATCC‐3^′^) [12]. Indexed PCR products were purified with AMPure XP beads (Beckman Coulter), quantified, and verified for fragment integrity using an Agilent 2100 Bioanalyzer. Libraries were pooled equimolarly and sequenced on an Illumina MiSeq platform (2 × 250 bp paired‐end).

### 2.3. Bioinformatics and Taxonomic Profiling

Raw sequence reads were processed using QIIME 2 (version 2024.2) [13]. Quality filtering and chimera removal preceded clustering of sequences into operational taxonomic units (OTUs) at 97% similarity using the SILVA 138.1 reference database for taxonomic assignment [14]. OTU tables were generated for downstream diversity and composition analyses.

Alpha (*α*)‐diversity indices (Shannon, Chao1) and beta (*β*)‐diversity (Bray–Curtis dissimilarity) were computed [15]. Taxonomic composition and relative‐abundance plots were visualized using the phyloseq and ggplot2 packages in R (version 4.3.2) [16]. Genera representing <0.5% relative abundance were grouped as “Others.”

To avoid overinterpretation due to 16S resolution limits, taxa within the *Enterobacterales* order (including *Escherichia/Shigella*) were reported at the genus level, without clinical inference unless validated independently. While statistical analyses utilized quality‐checked data from all 15 sequenced samples, only the 13 samples processed in the main sequencing batch are displayed in figures for visual consistency.

### 2.4. Functional Prediction and KEGG Pathway Analysis

Functional metagenomic prediction was performed using PICRUSt2 (version 2.5.1) [17], normalizing for 16S rRNA copy number. Predicted genes were mapped to KEGG pathways [18], and results are presented as predicted functional potentials rather than measured expression.

Heatmap visualization of KEGG pathway abundances was generated in R using the pheatmap package with Euclidean distance and complete‐linkage clustering [16]. Relative abundances < 0.5% were grouped as “Others,” and zero values were replaced with half the smallest nonzero abundance before log transformation.

### 2.5. Statistical Analysis

Continuous variables were summarized as mean±standard deviation (SD). Microbial *α*‐diversity indices were described using summary statistics without between‐group comparisons. Associations between microbial relative abundances (ASVs or genera) and serum lipid parameters, including TGs, HDL‐C, LDL‐C, and TC were evaluated using Spearman’s rank correlation with the Benjamini–Hochberg false discovery rate (FDR) method for multiple testing correction [19]. Partial correlations were adjusted for age, sex, and body mass index (BMI); however, adjustment for additional metabolic and lifestyle confounders was not possible.

*β*‐Diversity patterns were visualized via principal‐coordinate analysis (PCoA) based on Bray–Curtis distances using QIIME 2 and R. All analyses were performed in R (version 4.3.2) and SPSS version 26.0 (IBM Corp., USA), with two‐tailed *p* < 0.05 considered significant before FDR adjustment.

## 3. Results

### 3.1. Participant Characteristics and Serum Lipid Profile

Fifteen adults with clinically established T2DM (11 males and 4 females) were included in the analysis. The mean ± SD age was 47.3 ± 9.6 years (range 29–61 years). Fasting lipid measures showed considerable interindividual variability: one participant (6.7%) had TC > 200 mg/dL, five (33.3%) had TGs > 150 mg/dL, 13 (86.7%) had HDL‐C < 50 mg/dL, and three (20%) had LDL‐C > 100 mg/dL, reflecting cohort characteristics rather than population prevalence (Table 1). Overall, the cohort displayed a mixed dyslipidemic profile characterized by elevated TGs and reduced HDL‐C, typical of insulin resistance in T2DM.

**Table 1:** Fasting serum lipid profile of study participants with type 2 diabetes mellitus.

Patient ID	Cholesterol (mg/dL)	Triglycerides (mg/dL)	HDL (mg/dL)	LDL (mg/dL)
P1	119	113	*51*	45
P2	123	70	*43*	66
P3	104	132	*36*	41
P4	179	*173*	*41*	*104*
P5	198	*210*	*51*	*105*
P6	*205*	*151*	73	*102*
P7	113	119	*43*	46
P8	106	70	*32*	60
P9	117	52	*47*	60
P10	162	*290*	*38*	67
P11	111	85	*38*	56
P12	116	146	*48*	41
P13	145	*261*	*36*	57
P14	151	135	*40.2*	83.8
P15	179	62	73	93

*Note:* Fasting serum lipid parameters for 15 participants (11 males and 4 females) are shown. Abnormal values are indicated in italics. The majority exhibited low HDL and moderate hypertriglyceridemia, consistent with diabetic dyslipidemia. Reference ranges (Cleveland Clinic, 2025): total cholesterol <200 mg/dL, triglycerides <150 mg/dL, HDL‐C ≥40 mg/dL (men), ≥50 mg/dL (women), ≥60 mg/dL considered protective, LDL‐C <100 mg/dL (<70 mg/dL for individuals with diabetes).

### 3.2. Gut Microbiota Composition

After quality filtering and denoising with DADA2, a mean of 58,400 ± 9800 high‐quality paired‐end reads per sample were retained. The community was dominated by Firmicutes, Actinobacteria, Bacteroidetes, and Proteobacteria, with minor Verrucomicrobia. Across participants, the F/B ratio tended to be elevated with relative Actinobacteria enrichment and Bacteroidetes depletion, consistent with a dysbiotic configuration reported in metabolic disease (no between‐group comparisons were performed).

**Figure 1:**
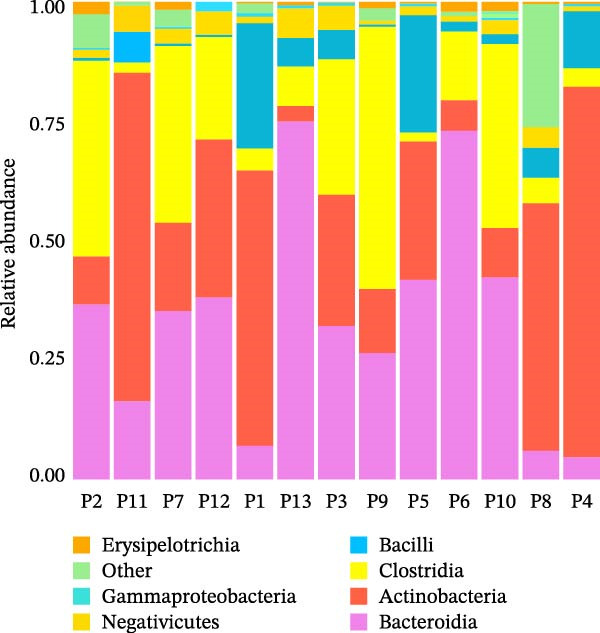
Species abundance bar plot showing taxonomic composition of fecal microbiota in participants with type 2 diabetes mellitus. Bar plot showing relative abundance of bacterial taxa identified by 16S rRNA sequencing in stool samples. Visualized for *n* = 13 samples due to sequencing‐run timing; all analyses used the full dataset (*n* = 15). Each bar represents one participant; taxa <0.5% are grouped as “Others.” A decrease in *Bacteroidia* and an increase in *Actinobacteria* and *Clostridia* indicate a Firmicutes‐ and Actinobacteria‐enriched, dysbiotic gut profile.

**Figure 2:**
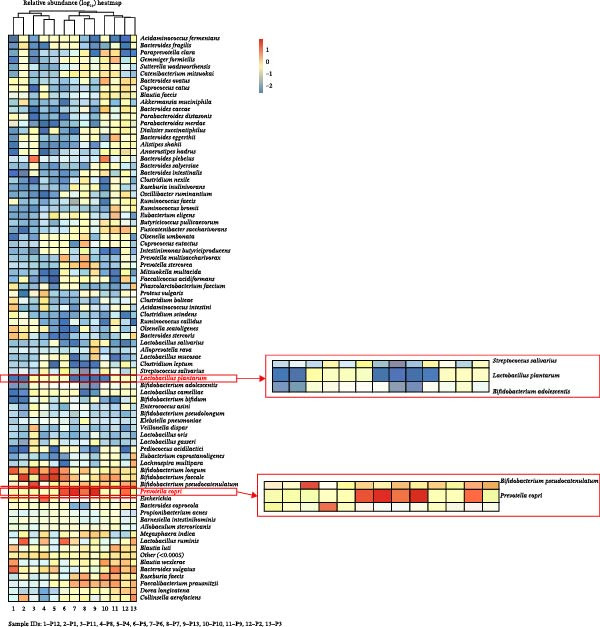
Heatmap of relative bacterial abundance in fecal samples from participants with type 2 diabetes mellitus. The heatmap displays species‐level relative abundance from 16S rRNA sequencing. Color intensity ranges from red (high abundance) to blue (low abundance). Most samples showed an increase in *Prevotella copri* and a decrease in *Lactobacillus plantarum*, reflecting a dysbiotic and proinflammatory microbial profile. Visualized for *n* = 13 samples due to sequencing‐run timing; all analyses used the full dataset (*n* = 15).

Genus‐level inspection suggested higher *Ruminococcus*, *Collinsella*, and *Bifidobacterium* in participants with higher TGs, and relatively lower *Akkermansia* and *Lactobacillus* in those with lower HDL‐C. These compositional tendencies are visualized in the stacked class‐level profiles showing reduced Bacteroidia and increased Actinobacteria/Clostridia (Figure 1) and in the species‐annotated heatmap (reported at genus level for Enterobacterales to respect 16S resolution limits) highlighting higher *Prevotella* (including the *P. copri* signal) and lower *Lactobacillus* (including the *L. plantarum* signal) across many samples (Figure 2). Summary listings of taxa with higher or lower relative abundance per participant are provided in Tables 2 and 3 (*Enterobacterales* are reported at the genus/order level without clinical inference). Species‐level labels within *Enterobacterales* are inferred from 16S rRNA data and should be interpreted as putative taxonomic signals rather than confirmed species identification.

**Table 2:** Bacterial taxa with increased relative abundance in fecal samples of participants with type 2 diabetes mellitus.

Patient ID	Increased species (relative abundance)
P1	*Bifidobacterium faecale and Lactobacillus ruminis*
P2	*Prevotella copri*
P3	*Lactobacillus ruminis*, *Collinsella aerofaciens*, *Bifidobacterium pseudocatenulatum*, and *Bacteroides vulgatus*
P4	*Bifidobacterium longum* and *Bifidobacterium faecale*
P5	*Prevotella copri and Lactobacillus ruminis*
P6	*Prevotella copri*
P7	*Prevotella copri*
P8	*Bifidobacterium faecale and Escherichia coli*
P9	*Roseburia faecis*, *Blautia wexlerae and Bifidobacterium faecale*
P10	*Bacteroides plebeius*
P11	*Bifidobacterium pseudocatenulatum and Bifidobacterium longum*
P12	*Bifidobacterium longum and Bacteroides vulgatus*
P13	*Prevotella copri*
P14	*Faecalibacterium prausnitzii and Butyricicoccus pullicaecorum*
P15	*Salmonella enterica*, *Shigella boydii*, and *Escherichia coli*

*Note:* This table summarizes bacterial taxa showing increased relative abundance in stool samples from 15 participants, based on 16S rRNA gene sequencing. Each patient ID represents one sample. Notable increases were observed in *Prevotella copri*, *Bifidobacterium longum*, *Lactobacillus ruminis*, and *Collinsella aerofaciens*, taxa associated with altered carbohydrate and lipid metabolism.

**Table 3:** Bacterial taxa showing lower relative abundance compared to literature‐reported eubiotic adult gut profiles.

Patient ID	Taxa showing reduced relative abundance
P1	*Fusicatenibacter saccharivorans*, *Proteus vulgaris*, *Lactobacillus plantarum*, *Bifidobacterium bifidum*, and *Pediococcus acidilactici*
P2	*Pediococcus acidilactici*, *Lactobacillus salivarius*, *Clostridium scindens*, *Acidaminococcus fermentans*, *Bacteroides fragilis*, and *Paraprevotella clara*
P3	*Ruminococcus callidus and Paraprevotella clara*
P4	*Parabacteroides merdae*, *Mitsuokella multacida*, *Faecalicoccus acidiformans*, *Bacteroides stercoris*, *Clostridium leptum*, and *Pediococcus acidilactici*
P5	*Lachnospira multipara*, *Ruminococcus callidus*, *Olsenella scatoligenes*, *Bacteroides stercoris*, *Akkermansia muciniphila*, and *Parabacteroides distasonis*
P6	*Lactobacillus plantarum*, *Clostridium leptum*, *Lactobacillus mucosae*, *Lactobacillus salivarius*, and *Dialister succinatiphilus*
P7	*Lactobacillus camelliae*, *Lactobacillus plantarum*, and *Clostridium leptum*
P8	*Bacteroides fragilis*, *Parabacteroides merdae*, *Dialister succinatiphilus*, *Anaerostipes hadrus*, *Oscillibacter ruminantium*, and *Mitsuokella multacida*
P9	*Bifidobacterium bifidum*, *Lactobacillus mucosae*, *Mitsuokella multacida*, *Faecalicoccus acidiformans*, and *Acidaminococcus fermentans*
P10	*Lactobacillus plantarum*, *Ruminococcus callidus*, *Intestinimonas butyriciproducens*, *Ruminococcus faecis*, *Clostridium nexile*, and *Roseburia inulinivorans*
P11	*Coprococcus cactus*, *Bacteroides caccae*, *Parabacteroides distasonis*, and *Clostridium nexile*
P12	*Acidaminococcus fermentans*, *Gemmiger formicilis*, *Bacteroides intestinalis*, *Olsenella umbonata*, *Lactobacillus plantarum*, *Lactobacillus camelliae*, *Bifidobacterium bifidum*, and *Pediococcus acidilactici*
P13	*Pediococcus acidilactici*, *Lactobacillus plantarum*, *Clostridium leptum*, *Acidaminococcus fermentans*, and *Paraprevotella clara*
P14	*Ruminococcus gnavus*, *Bacteroides acidifaciens*, and *Parabacteroides distasonis*
P15	—

*Note:* Bacterial taxa with reduced abundance compared to literature‐reported eubiotic gut microbiota patterns (no internal control group was included). Notable loss of *Lactobacillus plantarum*, *Akkermansia muciniphila*, *Bacteroides fragilis*, and *Parabacteroides distasonis* indicates diminished SCFA production and barrier function.

Because this was a single‐arm cohort without an internal healthy comparator, references to depletion or enrichment are contextualized relative to literature‐reported eubiotic adult microbiota profiles rather than direct experimental controls.

### 3.3. Microbial Diversity Indices

Alpha diversity (Shannon, Chao1) indicated modest richness (mean Shannon = 3.42 ± 0.38; Chao1 = 210 ± 54), generally lower than eubiotic adult references. Numeric richness values by sample are provided in Table 4 (reported as OTU counts clustered at 97% similarity). Bray–Curtis *β*‐diversity showed marked interindividual heterogeneity without clear clustering by lipid categories, consistent with individualized dysbiosis patterns (distribution of sample‐wise relative abundances shown in Figures 1 and 2).

**Table 4:** Operational taxonomic unit (OTU) counts indicating microbial richness and diversity in fecal samples of participants with type 2 diabetes mellitus.

Sample name	Tag number	OTU number
P1	55471	303
P2	47626	317
P3	48337	314
P4	57618	259
P5	56073	276
P6	56095	294
P7	47940	320
P8	53929	275
P9	43484	301
P10	45470	273
P11	57402	273
P12	53282	265
P13	54013	294

*Note:* This table shows OTU counts derived from 16S rRNA gene sequencing of stool samples from 15 participants with type 2 diabetes mellitus. OTUs were generated by clustering sequences at 97% similarity, representing distinct bacterial taxa within each sample. Figures display *n* = 13 samples due to sequencing‐run timing; two pilot samples processed earlier using identical protocols were excluded from these visualization panels for consistency. All numerical analyses and diversity metrics were performed on the full dataset (*n* = 15).

### 3.4. Taxa Associated With Lipid Parameters

**Figure 3:**
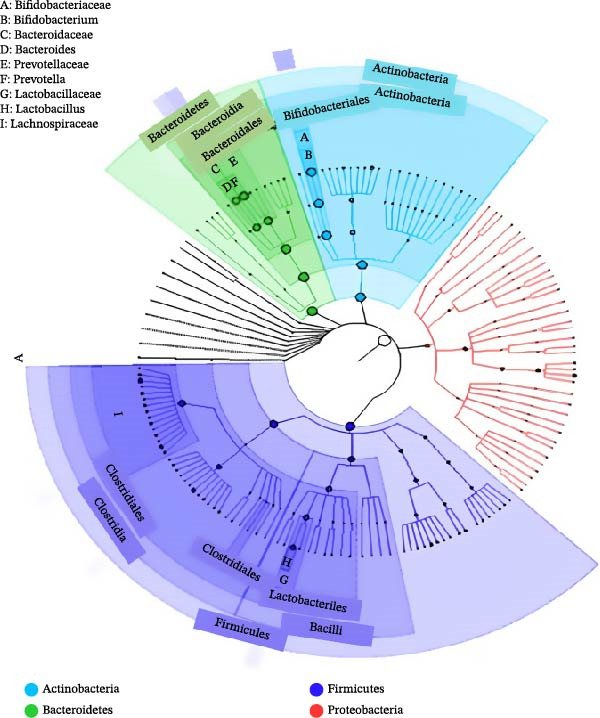
GraPhlAn phylogenetic map of gut microbiota in participants with type 2 diabetes mellitus. The circular cladogram shows the phylogenetic composition of gut bacteria. Firmicutes and Actinobacteria were dominant, while Bacteroidetes were reduced, indicating a Firmicutes‐rich, dysbiotic microbiome in diabetic subjects.

Within‐cohort correlations (Spearman *ρ*; FDR‐adjusted *p*  < 0.10) indicated: (a) TGs positively associated with *Collinsella* (ρ = 0.58) and *Ruminococcus* (*ρ* = 0.46), and negatively with *Akkermansia* (*ρ* = −0.51); (b) HDL‐C positively associated with *Akkermansia* and *Faecalibacterium*, and negatively with *Collinsella*; and (c) LDL‐C showed weak positive trends with *Bifidobacterium* and *Clostridium sensu stricto 1*. These associations align with the abundance patterns visualized in Figure 2 (higher *Prevotella* and *Collinsella*, lower *Lactobacillus* signal in several participants) and the community structure emphasized in Figures 1 and 3. Results are associative and not causal.

### 3.5. Relative Abundance Patterns

Genus‐level bar plots demonstrated recurring features across individuals: relative depletion of *Bacteroides* and *Parabacteroides* (key SCFA‐linked lineages); enrichment of *Ruminococcus* and *Collinsella* (taxa associated with energy harvest and bile‐acid transformation); and reduced *Akkermansia muciniphila*, a mucin‐degrading commensal supporting barrier integrity (Figure 1; participant‐level details in Tables 2 and 3). Together these shifts suggest a community configuration favoring lipid accumulation and proinflammatory signaling.

### 3.6. Phylogenetic Structure

The GraPhlAn cladogram highlighted dense central nodes for Firmicutes and Actinobacteria with sparser peripheral Bacteroidetes, underscoring a Firmicutes‐weighted architecture typical of metabolic dysbiosis. Prominent core contributions from *Ruminococcus*, *Bifidobacterium*, and *Collinsella* contrasted with underrepresentation of *Akkermansia* and *Bacteroides* (Figure 3).

### 3.7. Predicted Functional Profiles

**Figure 4:**
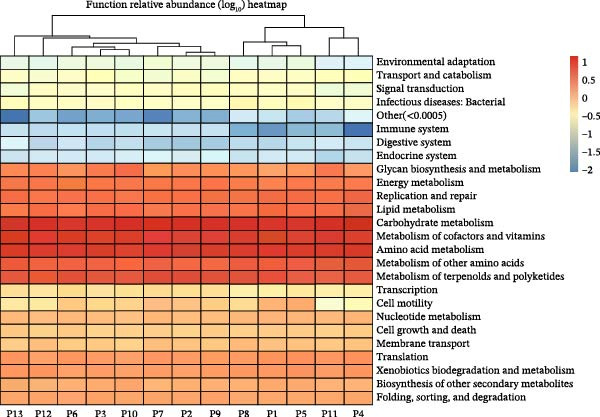
KEGG (Kyoto Encyclopedia of Genes and Genomes) pathway heatmap of collected stool samples. The heatmap illustrates predicted microbial functional pathways in diabetic participants, showing upregulation of lipid, amino acid, and carbohydrate metabolism and downregulation of immune and digestive pathways, reflecting metabolic dysregulation within the gut microbiome. Visualized for *n* = 13 samples due to sequencing‐run timing; all analyses used the full dataset (*n* = 15).

PICRUSt2‐based inference suggested higher predicted potential in pathways related to lipid and carbohydrate metabolism and secondary bile acid biosynthesis, with comparatively lower potential in vitamin biosynthesis, mucin‐related functions, and immune‐modulatory pathways (Figure 4). These are computational predictions (not direct metagenomic measurements) and should be interpreted as indicative functional tendencies consistent with the compositional shifts in Figures 1–3.

### 3.8. Integrated Interpretation

Taken together, the cohort exhibited a low‐diversity, Firmicutes/Actinobacteria‐weighted microbiome with relative loss of SCFA‐linked and barrier‐supportive taxa. Higher TGs and lower HDL‐C co‐occurred with enrichment of *Collinsella*/*Ruminococcus* and depletion of *Akkermansia*/*Faecalibacterium* (patterns summarized in Tables 2 and 3 and visualized in Figures 1–3). Predicted KEGG functions (Figure 4) supported an increased microbial capacity for lipid/carbohydrate metabolism, consistent with enhanced gut–liver cross‐talk in diabetic dyslipidemia.

## 4. Discussion

### 4.1. Principal Findings

In adults with T2DM, we observed a dyslipidemic pattern characterized by low HDL‐C and frequent hypertriglyceridemia alongside reduced gut microbial diversity and a Firmicutes/Actinobacteria‐weighted community. Taxonomic shifts included relative depletion of barrier‐ and SCFA‐linked taxa (e.g., *Akkermansia muciniphila*, *Faecalibacterium prausnitzii*, and *Lactobacillus* spp.) with enrichment of *Collinsella*, *Ruminococcus*, and *Prevotella* signals. Predicted functional capacities suggested higher potential for lipid/carbohydrate and secondary bile acid pathways and comparatively lower potential in barrier/immune‐supportive functions, consistent with a metabolically activated, proinflammatory intestinal milieu [1, 2, 4–6, 8–10, 17, 18].

### 4.2. Context Within Existing Literature

Multiple lines of evidence link gut composition and function with circulating lipids and insulin sensitivity. Inter‐individual variation in blood lipids is partly explained by microbiome structure and function [20], while integrative omics implicate *Prevotella copri* in insulin resistance through BCAA biosynthesis and bile acid interactions [21–23]. Cardiometabolic cohorts have also associated *Collinsella* enrichment and broader community alterations with atherosclerosis and atherogenic lipid signatures [24, 25]. Conversely, *A. muciniphila*, a mucin‐degrading commensal linked to improved metabolic profiles in dietary interventions, has been proposed as a next‐generation adjunct for metabolic disease modulation [26, 27]. These literature trends align with the compositional and predicted functional features we observed in T2DM, in which lipid absorption/transport and hepatic handling are influenced by microbially transformed bile acids and SCFA‐mediated signaling to AMPK/PPAR pathways [1, 2, 4–6, 18].

### 4.3. Mechanistic Alignment

#### 4.3.1. SCFAs and Epithelial Barrier

Butyrate enhances tight‐junction assembly and epithelial integrity, supporting anti‐inflammatory signaling and limiting endotoxemia that can exacerbate hepatic lipogenesis and dyslipidemia [28]. The relative loss of SCFA‐associated taxa in our cohort is coherent with impaired barrier and metabolic signaling [1, 4–6, 28].

#### 4.3.2. Bile‐Acid Signaling

Microbial BSH activity and downstream conversions shape FXR/TGR5 signaling, with downstream effects on cholesterol synthesis, VLDL secretion, and HDL remodeling [2, 5, 6, 18]. The enrichment of predicted lipid/carbohydrate pathways in our data is compatible with a strengthened gut–liver axis that may favor atherogenic lipid profiles in T2DM [2, 5, 6, 18].

#### 4.3.3. Beyond the F/B Ratio

Although an elevated F/B ratio appeared, it is an imprecise biomarker across diets, medications, and geographies [1]. Species‐ and pathway‐level readouts (e.g., *Collinsella*, *Akkermansia*, and *P. copri*; SCFA, and bile‐acid modules) offer higher mechanistic specificity for metabolic risk stratification [1, 2, 4–6, 20–27].

### 4.4. Clinical and Translational Implications

Microbiota‐informed adjuncts may complement standard lipid management in T2DM.

1. Dietary fiber/prebiotics to restore SCFA production and barrier function [1, 4–6, 23, 28].
2. Targeted probiotics/next‐generation biotherapeutics, including *A. muciniphila* and selected *Lactobacillus/Bifidobacterium* strains, which have shown promise for metabolic endpoints [9, 26, 27].
3. Bile‐acid‐centric strategies that modulate FXR/TGR5 signaling via microbial pathways (dietary or therapeutic) [2, 5, 6, 18].
4. Integration with lipid‐lowering therapy. Microbiome composition and function may help explain inter‐individual variability in lipid responses and could be leveraged in precision approaches [5].

### 4.5. Strengths and Limitations

Strengths include paired serum–stool profiling and convergence between taxonomic and predicted functional signals, with conservative reporting at the genus level where 16S resolution is limited. Limitations include modest sample size, cross‐sectional design, absence of a matched nondiabetic comparator, and reliance on 16S‐based inference rather than shotgun metagenomics and direct metabolomics. Future work should incorporate longitudinal and interventional designs with shotgun metagenomics, targeted metabolomics (SCFAs, bile acids, and BCAAs), and robust multivariable modeling to confirm biomarkers and mechanisms [2, 5, 6, 18, 20–23, 26–28].

The high prevalence of low HDL‐C observed in this cohort may reflect sampling characteristics rather than population‐level estimates and should not be generalized beyond the study group.

Given the cross‐sectional design, this study cannot determine whether gut microbial alterations precede dyslipidemia, arise as a consequence of lipid abnormalities, or reflect shared upstream determinants such as diet, glycemic control, or medication exposure. Longitudinal and interventional studies will be required to establish temporal directionality and mechanistic causation.

## 5. Conclusion

In adults with T2DM, this pilot study identified associative microbial patterns characterized by reduced microbial diversity, enrichment of Firmicutes and Actinobacteria, and depletion of SCFA‐linked and barrier‐supportive taxa. Within‐cohort analyses showed that higher triglyceride levels and lower HDL‐C were associated with increased abundance of *Collinsella* and *Ruminococcus*, alongside depletion of *Akkermansia muciniphila* and *Faecalibacterium*. These microbial shifts are consistent with an association between gut microbial dysbiosis and a gut–liver metabolic axis in diabetic dyslipidemia.

Functional predictions (PICRUSt2) revealed enhanced microbial potential for lipid, carbohydrate, and secondary bile acid metabolism, together with reduced immune and mucin‐associated pathways, suggesting a metabolically activated, proinflammatory intestinal environment. Collectively, these findings illustrate a biologically plausible link between microbial composition, metabolic function, and lipid regulation in T2DM.

Clinically, the results suggest hypothetical adjunctive strategies that warrant evaluation in controlled trials to conventional lipid management, such as dietary fermentable fiber, targeted probiotics (*Akkermansia*, *Lactobacillus*, and *Bifidobacterium*), and bile‐acid‐modulating strategies—to improve metabolic outcomes.

The study’s limitations include the small sample size, absence of a nondiabetic control group, and inherent taxonomic resolution constraints of 16S rRNA sequencing. Future research should employ larger, longitudinal, and interventional multiomics designs integrating metagenomics, metabolomics, and advanced statistical modeling to validate these associations and clarify causality. Confirmatory evidence could justify incorporating microbiome‐guided nutritional and therapeutic strategies into comprehensive lipid management for individuals with T2DM.

These findings should be viewed as hypothesis‐generating and intended to inform the design of larger, controlled, multiomics studies.

## Author Contributions

Conceptualization: Godfred Antony Menezes and Priyadharshini Sekar. Methodology: Godfred Antony Menezes, Priyadharshini Sekar, Areebah Akhter, and Ketaki Devendra Tayade. Investigation (patient recruitment and sample handling): Areebah Akhter, Ketaki Devendra Tayade, Sana Fathima, Zaina Falak Zahir Hussain, and Abhay Nigam. Resources (clinical access, sample provision, and patient data): Abhay Nigam. Software and bioinformatics: Priyadharshini Sekar and Godfred Antony Menezes. Visualization: Areebah Akhter, Ketaki Devendra Tayade, Sana Fathima, and Zaina Falak Zahir Hussain. writing – original draft preparation: Godfred Antony Menezes, Areebah Akhter, and Ketaki Devendra Tayade. writing – review and editing: Priyadharshini Sekar, Godfred Antony Menezes, Sana Fathima, Zaina Falak Zahir Hussain, and Abhay Nigam.

## Funding

This research received no external funding.

## Ethics Statement

The study was conducted in accordance with the Declaration of Helsinki and approved by the Ras Al Khaimah Research and Ethics Committee (RAK‐REC) in Ras Al Khaimah, accredited by the UAE’s Ministry of Health and Prevention (MOHAP), United Arab Emirates.

## Consent

Informed consent was obtained from all subjects involved in the study prior to enrollment and sample collection.

## Conflicts of Interest

The authors declare no conflicts of interest.

## Data Availability

Processed datasets and analysis scripts are available from the corresponding author upon reasonable request.
